# Supplementary material for: Change point detection with multiple alternatives reveals parallel evaluation of the same stream of evidence along distinct timescales
Source: Sci Rep. 2021 Jun 23;11:13098. doi: 10.1038/s41598-021-92470-y (PMC8222317; doi:10.1038/s41598-021-92470-y)
Supplement: Supplementary file 1 — Supplementary Information. [file 41598_2021_92470_MOESM1_ESM.pdf]

Hit Rate Threshold P-Values

Subject	Increase	Decrease
**1**	0.004	<.001
**2**	<.001	<.001
**3**	<.001	<.001
**4**	<.001	<.001
**5**	<.001	<.001
**6**	<.001	<.001
**7**	<.001	<.001
**8**	0.001	<.001
**9**	<.001	<.001
**Combined**	<.001	<.001

Supplementary Table S1*. Hit rate threshold p-values. P-values for comparisons of informed versus uninformed condition hit rate thresholds for the increase and decrease detections were calculated via bootstrap for all subjects.*

Reaction Time P-Values

Subject	P-Value
**1**	<.001
**2**	<.001
**3**	<.001
**4**	0.004
**5**	<.001
**6**	<.001
**7**	0.021
**8**	0.017
**9**	0.001
**Combined**	<.001

Supplementary Table S2*. Reaction time p-values. P-values for comparisons of informed versus uninformed condition reaction times were calculated via bootstrap for all subjects.*

False Alarm Difference P-Values

Subject	Increase	Decrease
**1**	0.015	0.015
**2**	<.001	<.001
**3**	<.001	<.001
**4**	0.136	<.001
**5**	<.001	0.004
**6**	<.001	<.001
**7**	<.001	<.001
**8**	0.725	0.014
**9**	<.001	<.001
**Combined**	<.001	<.001

Supplementary Table S3*. False alarm rate differences p-values. P-values for comparisons of informed versus uninformed condition false alarm rate differences for the increase and decrease detections were calculated via bootstrap for all subjects.*

False Alarm Number of Clicks From Baseline P-Values

Subject	Increase	Decrease
1	0.092	0.044
2	0.383	0.459
3	0.299	0.013
4	0.007	0.003
5	0.985	0.742
6	0.076	<.001
7	0.002	<.001
8	0.317	0.003
9	0.011	0.08
Combined	<.001	<.001

Supplementary Table S4. *False alarm number of clicks from baseline p-values. P-values for comparisons of informed versus uninformed condition false alarm rate number of clicks for the increase and decrease detections were calculated via bootstrap for all subjects.*

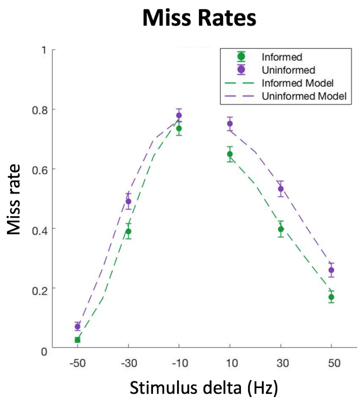

Supplementary Figure S5. *Miss rates. Experimental combined subject data with miss rate plotted as a function of change in click rates compared to the estimated model performance. Error bars indicate 95% confidence intervals. N= 9 subjects.*

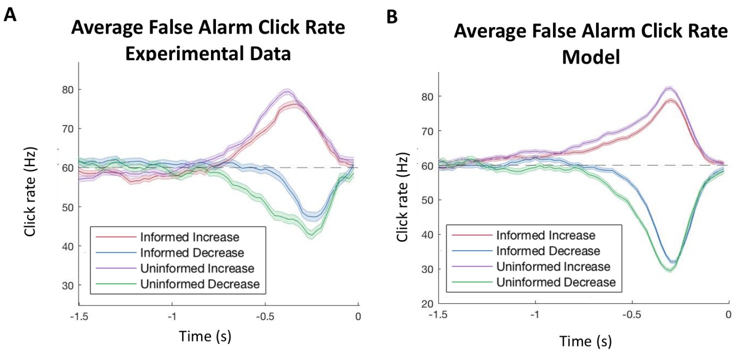

Supplementary Figure S6. *False alarm RC kernels. A) Detection kernels of different trial types for combined subject data. B) Detection kernels of different trial types for model fit. Shaded*

regions indicate the standard error of the mean. Dashed line indicates baseline click rate. Data is combined from all subjects. N=9 subjects.

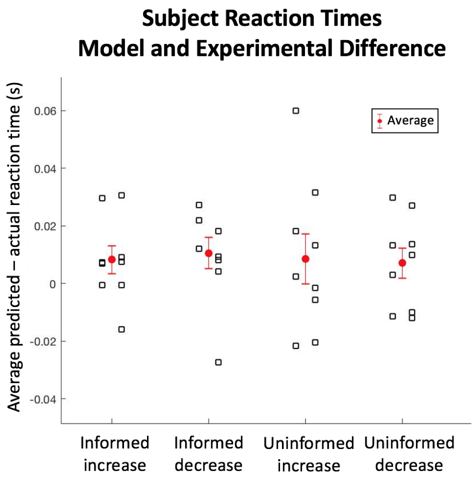

Supplementary Figure S7. *Model reaction time predictions. The difference between the average reaction times as predicted by the model fit and the experimental reaction times for each trial condition. Error bars indicate the standard error of the mean. N=9 subjects.*

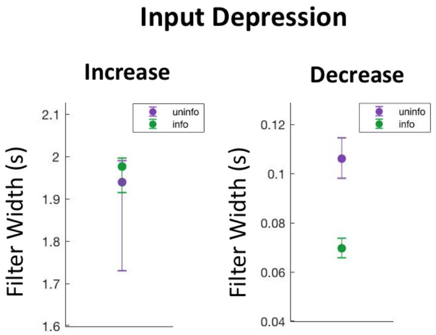

Supplementary Figure S8. *Model filter width including input depression. Detection filter widths for informed and uninformed conditions when input depression was incorporated into the model. Input depression used a magnitude of 0.45 and time constant of 20 ms, the strongest depression found from human subjects in a Poisson clicks task in Brunton 2013.*

Model Parameter Statistics

Parameter	Increase			Decrease		
	P-Value	T-Statistic	Degrees of Freedom	P-Value	T-Statistic	Degrees of Freedom
Filter Width	0.005	2.83	8353	<.001	8.49	8402
Bound	<.001	8.75	8353	<.001	4.26	8402
Mean NDT	0.71	0.37	8353	0.023	2.27	8402
Noise	0.03	2.17	8353	<.001	8.70	8402

Supplementary Table S9. *Model parameter statistics summary. The table shows the p-values, t-statistics, and degrees of freedom that went into the statistical comparisons for each of the model parameters.*

Recovered Model Parameters

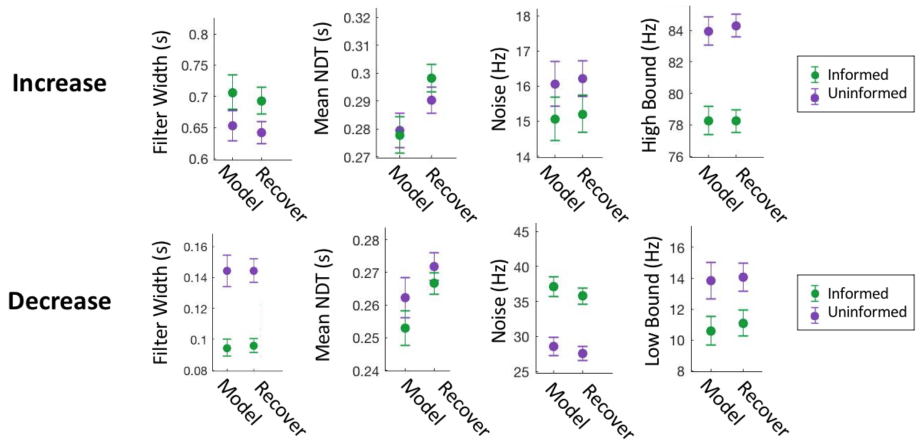

Supplementary Figure S10. *Recovery of model parameters from known values. Change detection parameters for model parameters recovered from synthetic data and model parameters fit to combined subject experimental data. All error bars indicate 95% confidence intervals. N=9 subjects.*
